# Pro-vegetarian dietary patterns and mortality by all-cause and specific causes in an older Mediterranean population

**DOI:** 10.1016/j.jnha.2024.100239

**Published:** 2024-04-20

**Authors:** Alejandro Oncina-Cánovas, Laura Torres-Collado, Manuela García-de-la-Hera, Laura María Compañ-Gabucio, Sandra González-Palacios, Antonio J. Signes-Pastor, Jesús Vioque

**Affiliations:** a Instituto de Investigación Sanitaria y Biomédica de Alicante, Universidad Miguel Hernández (ISABIAL-UMH), 03010 Alicante, Spain; b Unidad de Epidemiología de la Nutrición, Departamento de Salud Pública, Historia de la Ciencia y Ginecología, Universidad Miguel Hernández (UMH), 03550 Alicante, Spain; c CIBER Epidemiología y Salud Pública (CIBERESP), Instituto de Salud Carlos III, 28034 Madrid, Spain

**Keywords:** Pro-vegetarian, Dietary patterns, All-Cause mortality, Cardiovascular disease, Cancer

## Abstract

**Purpose:**

Pro-vegetarian (PVG) dietary patterns have shown health benefits, although the evidence concerning their association with mortality is scarce, particularly in older populations. We investigated the effect of three defined PVG patterns on all-cause, cardiovascular disease (CVD) and cancer mortality risk in an older Mediterranean population.

**Methods:**

We analysed baseline data from 597 adults aged 65 and older who participated in a population-based cross-sectional study, and mortality during a 12-year period. We used a validated food frequency questionnaire to estimate the adherence in tertiles to three evidence-based PVG dietary patterns: a general PVG pattern (gPVG) and two specific variations (healthful -hPVG, and unhealthful -uPVG). The gPVG pattern incorporated data from 12 food groups, consisting of 7 plant-based and 5 animal-based. The hPVG and uPVG versions included information from 18 food groups (4 food groups added and the splitting of 2 food groups). We used Cox regression to estimate hazard ratios (HR) and 95% confidence intervals (CI) adjusting for relevant covariates.

**Results:**

After the 12-years follow-up period, moderate adherence to hPVG pattern was associated with lower all-cause and CVD mortality whereas greater adherence to uPVG pattern was associated with higher all-cause and CVD mortality. Compared with those in the lowest tertile, participants in the second tertile of adherence to the hPVG pattern showed a significant lower risk of all-cause mortality (HR = 0.59; 95%CI: 0.43, 0.82) and CVD mortality (HR = 0.47; 0.28, 0.78). Participants in the highest tertile of adherence to the uPVG showed an increased mortality risk of all-cause (HR = 1.53; 1.07, 2.19) and CVD (HR = 2.10; 1.19, 3.70). No significant associations were found between adherence to any of the PVG dietary patterns and cancer mortality.

**Conclusion:**

Moderate adherence to a healthy PVG pattern reduced the long-term mortality risk for all-cause and CVD in an older Mediterranean population, while higher adherence to an unhealthy PVG pattern increased the risk of all-cause and CVD mortality.

## 1 Introduction

The increased life expectancy has led to a higher prevalence of non-communicable diseases, with cardiovascular disease (CVD) and cancer emerging as the most important causes of death [1]. Quitting smoking or not smoking, regular physical activity, and following a healthy diet are some of the habits that can help to reduce the risk of CVD and cancer, allowing individuals to live longer [2].

Regarding diet, the interest in the role of plant-based dietary patterns (PDI) has increased over the last decade for ethical, environmental and health reasons [3]. Although the vegetarian diets are the most popular PDI, prevalence of vegetarianism remains low [4]. Vegetarian patterns are characterized by the exclusion of animal foods, and this can lead to fewer followers due to concerns about nutritional adequacy or a lack of awareness [5]. In this sense, pro-vegetarian (PVG) dietary patterns are more focused on prioritising plant foods rather than excluding animal foods [6]. Studying these patterns provides the advantage of examining the gradual incorporation of plant-based foods. Additionally, since not all plant-based foods are equally recommendable, there may exist both healthful and unhealthful PVG patterns [7]. The evidence for these PVG dietary patterns supports beneficial associations with obesity [8,9], cardiometabolic risk [10] and different types of cancer [[11], [12], [13], [14]]. But the benefits of these patterns may go further, as there is some evidence that they may also reduce mortality risk. A previous study that included more than 100,000 South Korean adults, showed that a greater adherence to a PDI was associated with a 24% lower risk of all-cause mortality while a higher adherence to an unhealthful PDI (uPDI) was associated with a higher risk of all-cause, CVD and cancer mortality [15]. One study based on a sample of NHANES III only showed a statistically significant association for those participants with a healthful PDI (hPDI) score above the median and all-cause mortality [16]. Martínez-González et al. also studied the relationship between a general PVG (gPVG) dietary pattern and mortality in the PREDIMED study and showed that a higher adherence to this pattern in older people at high cardiovascular risk was associated with a 41% reduction of all-cause mortality [6].

Despite the evidence that these patterns may be related to mortality, the relationship is not fully consistent, especially in older Mediterranean populations. Thus, our aim was to evaluate the association between three PVG dietary patterns (general-gPVG, healthful-hPVG and unhealthful-uPVG) and the long-term mortality for all-cause, CVD and cancer in an older Mediterranean population.

## 2 Material and methods

### 2.1 Study design and population

We used data from participants in the European Eye Study (EUREYE) project in Spain, a population-based, cross-sectional study conducted in seven European countries in 2000–2001 [17]. The main objective of the study was to assess the prevalence of age-related macular degeneration, and risk factors among the elderly population (aged 65 years and older) in Europe. In the EUREYE-Spain study, a total of 597 subjects (54.3% females) were enrolled in the province of Alicante, Spain. All participants were interviewed at baseline using structured questionnaires to collect information on main lifestyle factors such as smoking, alcohol consumption, dietary habits and sociodemographic characteristics. All participants were informed of the aims of the study and agreed by informed consent to complete the interview. The study received ethical approval from the Local Ethics Committee of the Hospital de San Juan and Miguel Hernández University in Alicante, Spain (PM-EU, QLK6-CT-1999-02094).

### 2.2 Dietary intake and pro-vegetarian dietary patterns

Diet was assessed using a semi-quantitative food frequency questionnaire (FFQ) validated in adult population in Spain [18]. The FFQ included 131 food items, along with their standard portion sizes. Participants were asked to report their usual intake over the previous year, with nine frequency of consumption options, ranging from "never or <1 month" to " ≥6 times a day."

We developed three evidence-based *a priori* PVG patterns, including the gPVG pattern by Martínez-González [6], and the hPVG and uPVG variations based on the methodology proposed by Satija et al. [7]. These dietary patterns were constructed using information from 18 food groups, including vegetables, fruits, legumes, whole grains, refined grains, boiled potatoes, fries or chips, nuts, olive oil, tea and coffee, fruit juices, sugar-sweetened beverages, sweets and desserts, meat and meat products, animal fats, eggs, fish and seafood, and dairy. Table S1 outlines the specific items within each food group and the scoring criteria for each pattern. The creation of these PVG dietary patterns involved the following steps. First, the consumption of the 18 food groups in grams was adjusted for total energy intake using the residuals method [19]. Then, the calorie-adjusted consumption was divided into tertiles, assigning values from 1 to 3 based on the consumption of each food group. For the gPVG food pattern, 7 components from plant-based food groups received positive scores (3 for the highest intake): vegetables, fruits, legumes, grains (whole and refined grains), potatoes (boiled potatoes and fries or chips), nuts, and olive oil. On the other hand, 5 components from animal-based food groups were scored inversely: meat and other meat products, animal fats, eggs, fish and seafood, and dairy, with a value of 3 for the lowest consumption. In the hPVG and uPVG patterns, the grains group was further divided into whole and refined grains, and the potatoes group into fries or chips and boiled potatoes. Additionally, four groups (1. tea and coffee; 2. fruit juices; 3. sugar-sweetened beverages; and 4. desserts and sweets) were introduced in both patterns. In these patterns the animal-based food groups also scored in reverse. In addition, since these dietary patterns consider the healthiness of plant-based foods, whole grains and boiled potatoes scored as healthy foods (positively in the hPVG and reverse in the uPVG). While refined grains, fries or chips, fruit juices, sugar-sweetened beverages, desserts and sweets scored as unhealthy foods (reversed in the hPVG and positive in the uPVG). All other plant-based food groups (vegetables, fruit, legumes, nuts and olive oil) scored as healthy foods.

To calculate the final score for each participant, we summed the points for the 12 (for gPVG pattern) or 18 (for hPVG and uPVG patterns) components. The possible scores ranged from 12 points (indicating minimum adherence) to 36 points (indicating maximum adherence) for the gPVG pattern, and from 18 points (minimum adherence) to 54 points (maximum adherence) for the hPVG and uPVG patterns.

### 2.3 Mortality assessment

Throughout the 12-year follow-up period, information regarding the cause and date of death was gathered from two sources: the Mortality Registry in the Valencian Region and the National Death Index from the Spanish Statistical Office. The cause of death was coded using the 10th version of the International Classification of Diseases (ICD-10). Deaths were grouped into three broad categories as follows: CVD (ICD-10: I00-I99), cancer (ICD-10 codes: C00-D49), and all-cause mortality. The latter category encompassed both CVD and cancer deaths, as well as deaths resulting from any other cause.

### 2.4 Other variables

Participants underwent interviews conducted by trained fieldworkers using structured questionnaires covering sociodemographic characteristics and lifestyle habits. Additionally, a health examination was carried out, including measurements of height and weight. Finally, the following variables were taken into account in our analysis: age (in years); sex (male; female); educational level (<primary or <7 years; primary or 7–10 years; >primary or >10 years); waist circumference (in cm, for male: normal: 78–94; moderate: 94–102; and large: >102; and for females: normal: 64–80; moderate: 80–88; and large: >88) [20]; sleeping time (hours/day); smoking habit (current; past; never); alcohol consumption (g/day); pre-existing chronic disease at baseline (self-reported diabetes, high blood cholesterol and hypertension); and TV watching (hours/day). Self-reported diseases in the elderly showed a high degree of agreement with the medical conditions recorded in their official health records [21,22].

### 2.5 Statistical analysis

We employed one-factor ANOVA for quantitative variables and chi-square tests for categorical variables to assess the differences in the baseline characteristics and lifestyles among the tertiles of adherence to the different PVG dietary patterns (gPVG, hPVG and uPVG). We labelled each level of adherence as: low, moderate and high.

To track each participant's progress, we calculated the person-years of follow-up from the baseline interview date in the study until the date of death or the completion of 12-year follow-up, whatever occurred first.

Furthermore, we explored the association between adherence to the different PVG dietary patterns and the risk of mortality during the 12-year follow-up period. We used Cox proportional regression models to estimate adjusted hazard ratios (HR) and 95% confidence intervals (CIs). Adherence to each pattern was assessed in tertiles. The first tertile (low adherence) was the reference. We adjusted two models. The first model was minimally adjusted for age and sex. The second model included additional variables previously identified as potential confounders in the literature, as well as variables that showed p-values <0.20 in the bivariate analysis. This model included: educational level (<primary or <7 years; primary or 7–10 years; >primary or >10 years) [23], waist circumference (normal; moderate; high) [24], sleeping time (hours/day) [25], smoking habit (current; past; never), alcohol consumption (grams/day), self-reported diabetes (no/yes), high cholesterol (no/yes), hypertension (no/yes) and TV-watching (hours/day) [26]. The association for every one-point increment in adherence to each PVG dietary pattern was also examined.

We used the Likelihood Ratio Test (LRT) to evaluate the overall significance of the association using the different PVG patterns. We estimated p-trend to explore dose-response relationship for the different PVG patterns adherence, considering these as continuous rather than categorical variables. We also generated cumulative incidence curves for tertiles of adherence to each PVG dietary pattern and all-cause mortality.

The statistical analyses were conducted using STATA, version 17® College Station, TX: StataCorp LP. A significance level of 0.05 was established.

## 3 Results

Baseline characteristics of participants according to tertiles of adherence to the three PVG dietary patterns are shown in Table 1. Participants with higher adherence to a hPVG dietary pattern were more likely to be younger and showed higher prevalence of self-reported diabetes and hypercholesterolemia. On the other hand, those participants with higher adherence to a uPVG dietary pattern were more likely to be male, smoker, with a higher alcohol consumption, higher hours of sleep, lower waist circumference and a lower prevalence of self-reported diabetes.

**Table 1:** Baseline characteristics of participants according to tertiles of the three PVG dietary patterns in the European Eye Study (EUREYE) (*n* = 597).

	gPVG^a^		
	Low <24 (n = 249)	Moderate 24−25 (*n* = 166)	High >25 (*n* = 182)
Sex, female (%)	57.8	52.4	51.1
Age (y)	74.0 (6.5)^b^	74.4 (6.9)	73.2 (5.6)
<Primary education (%)	52.6	59.0	52.2
Waist circumference, large^c^ (%)	66.9	67.1	57.7
Smoking, current smoker (%)	13.3	18.1	12.1
Alcohol intake (g/d)	7.2 (10.1)	7.9 (14.3)	7.1 (12.5)
TV watching (hours/day)	4.2 (1.8)	4.1 (2.1)	4.0 (1.8)
Sleep (hours/day)	7.9 (2.1)	8.1 (2.2)	8.0 (1.8)
Hypertension (%)	45.2	41.6	38.3
High blood cholesterol (%)	17.7	17.2	21.7
Diabetes (%)	19.7	17.5	19.9

	hPVG		
	Low <36 (*n* = 266)	Moderate 36−38 (*n* = 181)	High >38 (*n* = 150)
Sex, female (%)	54.1	52.5	56.7
Age (y)	**74.5 (6.8)**	**73.8 (6.2)**	**72.8 (5.6)**
<Primary education (%)	59.0	49.7	51.3
Waist circumference, large (%)	64.0	70.7	56.4
Smoking, current smoker (%)	13.6	15.5	14.0
Alcohol intake (g/d)	6.9 (9.9)	7.2 (11.5)	8.5 (15.8)
TV watching (hours/day)	4.2 (2.0)	4.0 (1.8)	4.0 (1.9)
Sleep (hours/day)	8.1 (2.1)	8.1 (1.9)	7.8 (1.9)
Hypertension (%)	47.1	39.1	36.7
High blood cholesterol (%)	**15.9**	**16.6**	**26.5**
Diabetes (%)	**14.3**	**20.4**	**26.0**

	uPVG		
	Low <35 (*n* = 214)	Moderate 35−38 (*n* = 219)	High >38 (*n* = 164)
Sex, female (%)	**65.4**	**57.5**	**35.4**
Age (y)	73.2 (5.9)	74.3 (6.6)	74.1 (6.6)
<Primary education (%)	54.7	53.0	55.5
Waist circumference, large (%)	**69.6**	**64.5**	**56.4**
Smoking, current smoker (%)	**11.7**	**13.2**	**19.1**
Alcohol intake (g/d)	**5.0 (7.8)**	**7.1 (13.0)**	**10.8 (14.6)**
TV watching (hours/day)	4.1 (1.7)	4.1 (2.0)	4.1 (2.0)
Sleep (hours/day)	**7.7 (1.9)**	**8.1 (2.0)**	**8.2 (2.1)**
Hypertension (%)	44.8	40.1	41.3
High blood cholesterol (%)	23.6	16.5	15.5
Diabetes (%)	**30.4**	**17.4**	**6.8**

Abbreviations: gPVG: general pro-vegetarian dietary pattern; hPVG, healthful pro-vegetarian dietary pattern; uPVG, unhealthful pro-vegetarian dietary pattern.

Bold values are p-value < 0.05.

a Comparisons of characteristics across tertiles of the PVG dietary patterns were performed by using 1-factor ANOVA for quantitative variables or chi-square tests for categorical variables.

b Mean (SD) (all such values).

c For male: >102 cm; and for females: >88 cm.

**Fig. 1:**
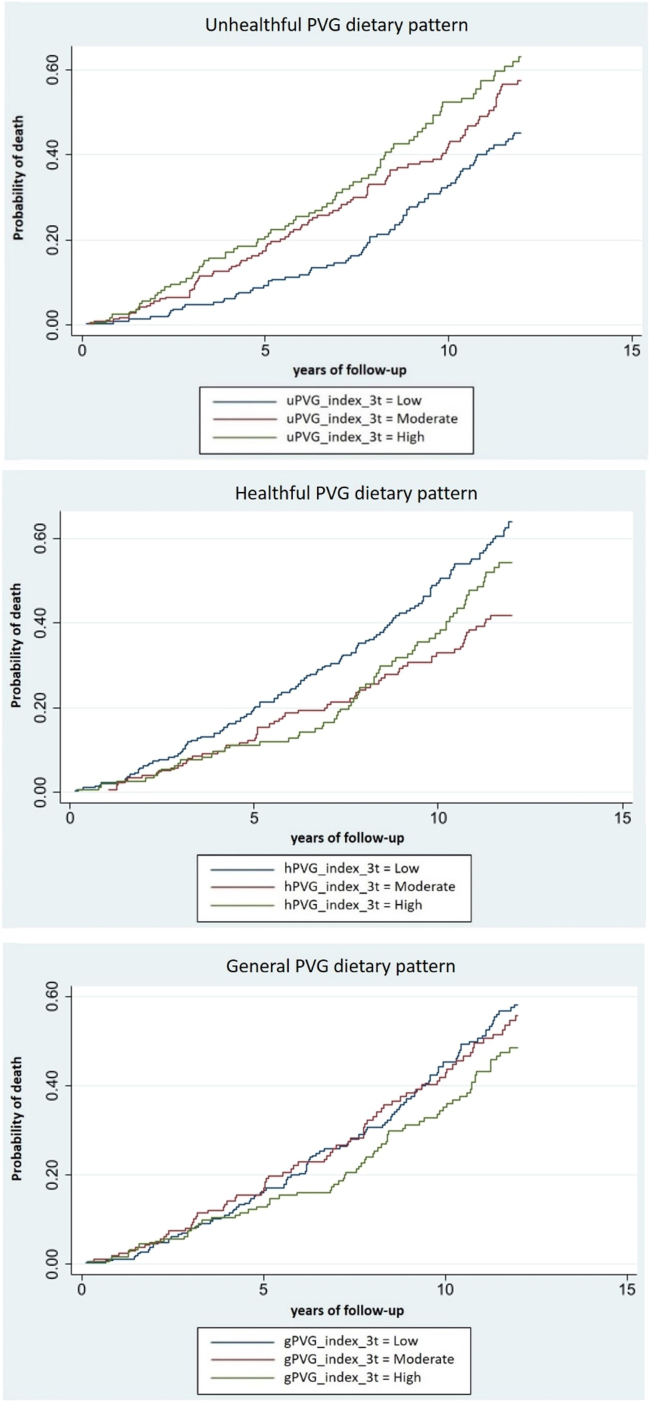
Curves of cumulative incidence for all-cause mortality during the study period according to tertile of adherence to the different PVG dietary patterns (gPVG, hPVG and uPVG) among elderly participants of EUREYE-Spain study (*n* = 597).

During the 12-year period (5813.9 persons-years), 251 deaths occurred, 100 (39.8 %) due to CVD and 58 (23.1 %) due to cancer. During the 12-year period, cumulative incidence curves for all-cause mortality were the lowest for a high adherence (T3) to a gPVG pattern, a moderate adherence (T2) to a hPVG and a low adherence (T1) to a uPVG (Fig. 1).

Table 2, Table 3 and 4 show the HRs for all-cause, CVD, and cancer mortality for each of the three PVG dietary patterns. The adherence to a gPVG was not significantly associated with all-cause or any specific cause of mortality (Table 2).

**Table 2:** Associations between adherence to a gPVG dietary pattern (in tertiles) and all-cause, cardiovascular disease and cancer mortality among participants of European Eye Study (EUREYE) (*n* = 597).

	gPVG					
	Low	Moderate	High	*p-*value^b^	*p-*trend^c^	Per 1 point increment
	*Follow-up at 12 years*					
All-cause, n (%)	249 (41.7)	166 (27.8)	182 (30.5)			
deaths, n	110	71	70			
person-years	2401.9	1590.9	1821.1			
HR (95% CI)						
Age and sex adjusted	1.00	0.79 (0.58−1.08)	0.87 (0.64−1.18)			
Multivariable^a^	1.00	0.78 (0.57−1.06)	0.85 (0.62−1.16)	0.256	0.238	0.96 (0.92−1.01)
						
CVD, n (%)	180 (40.4)	121 (27.1)	145 (32.5)			
deaths, n	41	26	33			
person-years	1924.8	1301.7	1583.8			
HR (95% CI)						
Age and sex adjusted	1.00	0.69 (0.42−1.14)	1.01 (0.64−1.60)			
Multivariable^a^	1.00	0.61 (0.35−1.05)	0.99 (0.61−1.62)	0.140	0.864	0.99 (0.92−1.07)
						
Cancer, n (%)	163 (40.4)	112 (27.7)	129 (31.9)			
deaths, n	24	17	17			
person-years	1826.5	1230.7	1442.4			
HR (95% CI)						
Age and sex adjusted	1.00	0.84 (0.43−1.63)	0.91 (0.49−1.72)			
Multivariable^a^	1.00	0.83 (0.43−1.62)	0.91 (0.48−1.71)	0.866	0.745	0.96 (0.88−1.05)

Abbreviations: gPVG: general pro-vegetarian dietary pattern; HR: hazard ratios; CI: confidence interval; CVD: cardiovascular disease.

a Cox regression model adjusted for age (in years), sex (male; female), educational level (<Primary or <7 years; Primary or 7–10 years; >Primary or >10 years), waist circumference (normal; moderate; large), sleeping time (hours/day), smoking habit (current; past; never), alcohol consumption (grams/day), self-reported diabetes (no/yes), high cholesterol (no/yes), hypertension (no/yes) and TV-watching (hours/day).

b p-value from LRtest.

c p-value from trend-test.

**Table 3:** Associations between adherence to a hPVG dietary pattern (in tertiles) and all-cause, cardiovascular disease and cancer mortality among participants of European Eye Study (EUREYE) (*n* = 597).

	hPVG					
	Low	Moderate	High	*p-*value^b^	*p-*trend^c^	Per 1 point increment
	*Follow-up at 12 years*					
All-cause, n (%)	266 (44.6)	181 (30.3)	150 (25.1)			
deaths, n	126	62	63			
person-years	2487.6	1825.2	1501.1			
HR (95% CI)						
Age and sex adjusted	1.00	**0.65 (0.48−0.88)**	0.93 (0.68−1.25)			
Multivariable^a^	1.00	**0.59 (0.43−0.82)**	0.90 (0.65−1.24)	**0.004**	0.209	0.98 (0.94−1.01)
						
CVD, n (%)	194 (43.5)	144 (32.3)	108 (24.2)			
deaths, n	54	25	21			
person-years	1981.6	1620.6	1208.1			
HR (95% CI)						
Age and sex adjusted	1.00	**0.54 (0.33−0.86)**	0.83 (0.50−1.40)			
Multivariable^a^	1.00	**0.47 (0.28−0.78)**	0.81 (0.47−1.39)	**0.009**	0.142	0.97 (0.92−1.03)
						
Cancer, n (%)	166 (41.1)	132 (32.7)	106 (26.2)			
deaths, n	26	13	19			
person-years	1846.6	1481.5	1171.5			
HR (95% CI)						
Age and sex adjusted	1.00	0.58 (0.30−1.14)	1.17 (0.65−2.13)			
Multivariable^a^	1.00	0.53 (0.26−1.06)	1.13 (0.60−2.14)	0.082	0.914	1.00 (0.94−1.08)

Abbreviations: hPVG: healthful pro-vegetarian dietary pattern; HR: hazard ratios; CI: confidence interval; CVD: cardiovascular disease.

Bold values are p-value < 0.05.

a Cox regression model adjusted for age (in years), sex (male; female), educational level (<Primary or <7 years; Primary or 7–10 years; >Primary or >10 years), waist circumference (normal; moderate; large), sleeping time (hours/day), smoking habit (current; past; never), alcohol consumption (grams/day), self-reported diabetes (no/yes), high cholesterol (no/yes), hypertension (no/yes) and TV-watching (hours/day).

b p-value from LRtest.

c p-value from trend-test.

We observed an inverse association between moderate adherence to a hPVG pattern and all-cause and CVD mortality throughout the follow-up period (Table 3). After 12-years of follow-up, compared to participants with lowest adherence (T1), those with moderate adherence (T2) to a hPVG dietary pattern showed a 41% (HR: 0.59; 95% CI: 0.43−0.82) and 53% (HR: 0.47; 95% CI: 0.28−0.78) lower risk of all-cause and CVD mortality in the adjusted model, respectively (Table 3). No statistically significant association was observed between the adherence to a hPVG and cancer mortality during the study period. When the adherence to a hPVG pattern was evaluated as a continuous term, we did not find any significant association.

Individuals with higher adherence (T3) to the uPVG dietary pattern showed a 53% higher risk of all-cause mortality (HR: 1.53; 95% CI: 1.07−2.19) when compared with those with the lowest adherence (T1) (Table 4). Furthermore, those with higher adherence (T3) to the uPVG pattern exhibited a 110% higher risk of CVD mortality (HR: 2.10; 95% CI: 1.19−3.70). No statistically significant association between adherence to an uPVG and cancer mortality was observed. Significant associations were found for all-causes and CVD mortality when the adherence to uPVG pattern was evaluated as continuous variable with a significant dose-response (*p*-trend < 0.05).

**Table 4:** Associations between adherence to a uPVG dietary pattern (in tertiles) and all-cause, cardiovascular disease and cancer mortality among participants of European Eye Study (EUREYE) (*n* = 597).

	uPVG					
	Low	Moderate	High	*p-*value^b^	*p-*trend^c^	Per 1 point increment
	*Follow-up at 12 years*					
All-cause, n (%)	214 (35.8)	219 (36.7)	164 (27.5)			
deaths, n	78	96	77			
person-years	2206.7	2093.8	1513.5			
HR (95% CI)						
Age and sex adjusted	1.00	1.21 (0.90−1.64)	1.24 (0.90−1.72)			
Multivariable^a^	1.00	1.31 (0.95−1.79)	**1.53 (1.07−2.19)**	0.056	**0.018**	1.02 (0.99−1.06)
						
CVD, n (%)	163 (36.5)	159 (35.7)	124 (27.8)			
deaths, n	27	36	37			
person-years	1842.1	1686.3	1281.9			
HR (95% CI)						
Age and sex adjusted	1.00	1.21 (0.73−2.01)	1.52 (0.89−2.59)			
Multivariable^a^	1.00	1.46 (0.86−2.47)	**2.10 (1.19−3.70)**	**0.036**	**0.010**	1.04 (0.99−1.10)
						
Cancer, n (%)	154 (38.1)	144 (35.7)	106 (26.2)			
deaths, n	18	21	19			
person-years	1744.0	1611.3	1144.3			
HR (95% CI)						
Age and sex adjusted	1.00	1.16 (0.62−2.18)	1.28 (0.65−2.50)			
Multivariable^a^	1.00	1.25 (0.63−2.48)	1.59 (0.77−3.31)	0.458	0.212	1.02 (0.95−1.09)

Abbreviations: uPVG: unhealthful pro-vegetarian dietary pattern; HR: hazard ratios; CI: confidence interval; CVD: cardiovascular disease.

Bold values are p-value < 0.05.

a Cox regression model adjusted for age (in years), sex (male; female), educational level (<Primary or <7 years; Primary or 7–10 years; >Primary or >10 years), waist circumference (normal; moderate; large), sleeping time (hours/day), smoking habit (current; past; never), alcohol consumption (grams/day), self-reported diabetes (no/yes), high cholesterol (no/yes), hypertension (no/yes) and TV-watching (hours/day).

b p-value from LRtest.

c p-value from trend-test.

## 4 Discussion

In this study, a higher adherence to healthful PVG dietary pattern was associated with lower all-cause and CVD mortality after a 12-year follow-up period. A higher adherence to an unhealthful PVG dietary pattern was associated to higher risk of all-cause and CVD mortality.

The adherence to the gPVG dietary pattern was not associated with the risk of all-cause and/or specific cause of mortality in our study. This lack of association with a gPVG has been also reported by other studies [16,27,28], although some studies have reported a protective association between adherence to this general pattern and the risk of all-cause [6,15,29] or specific mortality [30,31]. This inconsistency might be attributed in part to variations in the study population's characteristics like age of participants, since some studies included younger participants than in our study [16,28,30]. The lack of association found in our study could be also due to the small sample size and the limited statistical power (*n* = 597).

The moderate adherence to the hPVG was associated with a lower risk of all-cause and CVD mortality in our study after 12 years of follow-up. A study with more than 60,000 participants from the Nurses' Health Study and the Health Professionals Follow-Up Study, also found a 10% lower all-cause mortality risk in those participants with higher hPDI adherence. Furthermore, for each additional 10 points of adherence, the risk of CVD mortality decreased by 9% [27]. In another study conducted by Kim et al., which included more than 12,000 participants at higher cardiovascular risk from the Atherosclerosis Risk in Communities study, a higher adherence to the hPDI pattern was also associated with a reduced risk of all-cause (19%) and CVD-specific mortality (11%) [30]. Other studies conducted in Spain [32], UK [33] and US [34] have also reported similar findings regarding this pattern.

Several mechanisms could account for the observed protective association between adherence to the hPVG pattern and the reduced risk of all-cause and CVD mortality. This pattern and its opposite, the uPVG pattern, were developed based on information available in the scientific literature regarding the relationship between various plant-based foods and the risk of chronic diseases such as diabetes and CVD [7]. In this sense, the hPVG pattern has shown positive associations with the risk of diabetes [7,35], obesity [9], and cardiometabolic markers [10]. Some key components of this pattern, such as coffee or tea, fruits and vegetables, and nuts, may provide an explanation for our results by their association with reduced overall and CVD mortality rates [[36], [37], [38], [39], [40]]. These plant-based foods are abundant in essential minerals, vitamins, and other bioactive compounds, including polyphenols and dietary fibers, which may play a pivotal role in anticoagulant, anti-inflammatory, and antioxidant processes [[41], [42], [43]]. In addition, replacing animal proteins with plant-based proteins has been associated with beneficial effects on mortality [44], in part through its influence on insulin regulation, specifically in reducing Insulin Growth Factor-1 levels—a protein linked to a higher risk of mortality [45]. In this regard, it is important to note that in our study, the association was not observed at the highest level of adherence but rather at a moderate level of adherence. This non-linear association between adherence to hPVG and mortality risk has been also documented in a prior study [16]. It may be attributed to the L-shaped curve relationship observed in certain foods positively scored in this pattern, such as fruits and vegetables [46]. In other words, the decline in mortality risk associated with the consumption of these foods doesn't follow a linear trend but reaches a plateau at a specific level.

A higher adherence to uPVG was associated with an elevated risk of all-cause and CVD mortality in our study. This pattern assigns positive scores to certain foods linked to increased mortality, such as fried potatoes [47] or sugar-sweetened drinks [48], which may provide an explanation for our findings. Moreover, previous studies have consistently showed a positive association between high adherence to this pattern and the risk of all-cause and CVD mortality [15,27,29,31,33,49].

These results can be explained by various mechanisms related to adherence to uPVG and its impact on both all-cause and CVD mortality. First, the uPVG pattern incorporates processed and refined plant-based foods, potentially leading to a nutritionally deficient pattern, characterized by abundant low-quality fats, sugars, salt and excessive energy [50], which can contribute to chronic low-grade inflammation and oxidative stress in the body [51]. These processes are linked to the development and progression of cardiovascular diseases and overall mortality [52]. In this sense, the consumption of sodium and free sugars has been strongly linked to the development of high blood pressure [[53], [54], [55], [56]], a major contributor to CVD incidence and mortality [57,58]. Furthermore, the consumption of ultra-processed products, foods which are included in the uPVG pattern, has been associated with substantial increases in fat mass [59] and diabetes risk [60], potentially increasing CVD risk. Secondly, these products often lack dietary fibre and contain additives that may disrupt the composition and diversity of the gut microbiota [61]. Dysbiosis of the gut microbiota has been linked to various cardiovascular risk factors, including inflammation, obesity, and insulin resistance [62,63], thereby increasing the risk of cardiovascular mortality. Thirdly, some components of ultra-processed foods, such as refined sugars and unhealthy fats, can induce endothelial dysfunction, impairing vasodilation and promoting atherosclerosis, which are key contributors to cardiovascular mortality [64,65]. Finally, ultra-processed foods have not only been related with a higher susceptibility to various health conditions but also elevate the risk of all-cause mortality [[66], [67], [68], [69]].

We did not observe a significant association between adherence to any of the PVG dietary patterns and cancer mortality in our study. Some studies have shown that gPVG and hPVG adherence were related to a lower risk of digestive cancers [13,70] and lower cancer mortality [31,33], while higher adherence to uPVG was associated with a higher risk of cancer mortality [15,31,33]. The low number of cancer deaths (*n* = 58) observed in our study may have reduced the statistical power to identify significant associations.

Our study may have several limitations. The information about diet was self-reported, which could introduce some misclassification error. However, dietary assessment was performed by trained interviewers using a validated FFQ [19]. In addition, data collection was limited to a single dietary measurement at baseline, and we were unable to track changes in adherence to the three PVG patterns throughout the follow-up period. However, earlier research has indicated that dietary habits tend to remain consistent over time [71,72]. Therefore, evaluating diet at baseline in cohort studies involving adult populations may still serve as a valid approach for investigating long-term effects on the risk of non-communicable diseases or mortality. Although our study has limited sample size, particularly, to explore associations with specific causes of death, the 12-years follow-up period allowed us to accumulate a satisfactory number of deaths to detect significant associations between two PVG dietary patterns and all-cause and CVD mortality. Although we adjusted for known mortality risk factors, important lifestyles, and characteristics, other potential confounding variables could have influenced our results. Finally, we considered several foods with heterogeneous characteristics (eg, some sweets or desserts) as plant-based foods because their main ingredients are plants, although they may contain animal fats; this could cause some misclassification, although any inaccuracy should be non-differential.

The current study has also some strengths. We used standardized and validated questionnaires to gathering information on food intake, socioeconomic characteristics, and lifestyles from a well-defined and representative Spanish Mediterranean population aged 65 years and older. In addition, the 12-year follow-up period enabled us to identify long-term significant associations. The use of two derivations of the PVG pattern such as hPVG and uPVG allowed us to assess their impact on all-cause mortality, as well as on CVD and cancer mortality according to their healthfulness. Finally, some associations showed a dose-response relationship, providing additional robustness and support for our main results.

## 5 Conclusion

This study, carried out with an elderly Mediterranean population, suggests that PVG dietary patterns may influence the risk of all-cause and CVD mortality. Be adhered to a hPVG pattern that prioritizes the consumption of fruits, vegetables, nuts and other healthy fats such as olive oil, whole grains, and legumes may be recommended, as it may reduce the risk of all-cause and CVD mortality. Whereas, adhering to an uPVG that includes highly processed plant-based foods, such as fried potatoes or sugar-sweetened drinks, may increase the risk of both all-cause and CVD mortality. Further prospective studies with larger sample sizes and long follow-up periods are necessary to confirm these findings.

## Contributions

AO-C contributed to methodology, formal statistical analysis and writing first draft; JV contributed to methodology, visualization, supervision, support in statistical analysis and reviewing of manuscript; LT-C, MG-d-H, LC-G, SG-P and AJS-P made a critical revision of the manuscript for intellectual content and approved the final manuscript.

## Conflict of interests

The authors declare that they have no known competing financial interests or personal relationships that could have appeared to influence the work reported in this paper.

## Funding

This work was supported by the official Spanish Institutions for funding scientific biomedical research, CIBER Epidemiología y Salud Pública (CIBERESP) and Instituto de Salud Carlos III (10.13039/501100004587ISCIII), through the Fondo de Investigación para la Salud (FIS), which is co-funded by the 10.13039/501100008530European Regional Development Fund (FIS_PI20/00557), the 10.13039/501100003359Generalitat Valenciana (AICO/2021/347) and Instituto de Investigación Sanitaria y Biomédica de Alicante (ISABIAL), (UGP-23-069 / 2023-0310).
