# Supplementary material for: Pro-vegetarian dietary patterns and mortality by all-cause and specific causes in an older Mediterranean population
Source: J Nutr Health Aging. 2024 Apr 20;28(7):100239. doi: 10.1016/j.jnha.2024.100239 (PMC12433779; doi:10.1016/j.jnha.2024.100239)
Supplement: Supplementary file 1 [file mmc1.docx]

| **Table S1**. Scoring criteria for each PVG dietary patterns^1^. | | | | |
| --- | --- | --- | --- | --- |
| **Food groups** | **Included food items** | **gPVG^3^** | **hPVG** | **uPVG** |
| Plant food groups^2^ |  |  |  |  |
| 1. Vegetables | Spinach, cabbage, brussels sprouts, cauliflower, lettuce, tomatoes, onion, carrot, pumpkin, green beans, cucumber, eggplant, zucchini, peppers, asparagus, mushroom, garlic, leek, watercress, parsnip, artichoke, celery | Positive | Positive | Reverse |
| 2. Fruits | Oranges, mandarin, grapefruit, banana, apple, pear, strawberries, cherries, peaches, fresh figs, watermelon, melon, grapes, pineapple, lime, kiwi, canned fruit | Positive | Positive | Reverse |
| 3. Legumes | Lentils, chickpeas, beans, peas | Positive | Positive | Reverse |
| 4. Whole grains | Whole-grain bread | Positive | Positive | Reverse |
| 5. Refined grains | White bread, rolls, breakfast cereals, white rice, white pasta | Positive | Reverse | Positive |
| 7. Potatoes | Boiled potatoes | Positive | Positive | Reverse |
| 6. Fries and chips | French fries, potato chips | Positive | Reverse | Positive |
| 8. Nuts | Nuts | Positive | Positive | Reverse |
| 9. Olive oil | Olive oil | Positive | Positive | Reverse |
| 10. Tea and coffee | Caffeinated coffee, decaffeinated coffee, tea | Not scored | Positive | Reverse |
| 11. Fruit juices | Orange juice, other package fruit juices | Not scored | Reverse | Positive |
| 12. Sugar-sweetened beverages | Carbonated soft drinks: cola, orange, lemon | Not scored | Reverse | Positive |
| 13. Sweets and desserts | Chocolate cookies, croissant, cakes, chocolate, bonbons, cocoa powder, sugar | Not scored | Reverse | Positive |
| Animal food groups |  |  |  |  |
| 14. Meat/meat products | Chicken with or without skin, beef, pork, lamb, game meat (rabbit, quail, duck), liver of beef, pork or chicken, viscera, ham, hamburger, bacon | Reverse | Reverse | Reverse |
| 15. Animal fats for cooking or as a spread | Butter | Reverse | Reverse | Reverse |
| 16. Eggs | Eggs | Reverse | Reverse | Reverse |
| 17. Fish and other seafood | Fried fish, white fish, blue fish (sardines, tuna), salted fish (cod, anchovies), clams, mussels, oysters, squid, octopus, shellfish (prawns, lobster and similar) | Reverse | Reverse | Reverse |
| 18. Dairy products | Whole milk, skim or low-fat milk, condensed milk, low-fat and whole fat yoghurt, cottage cheese, curd, white or fresh cheese, creamy cheese or cheese in portions, cured or semi-cured cheese (Manchego), custard, flan, pudding, ice cream | Reverse | Reverse | Reverse |
| Abbreviations: gPVG, general pro-vegetarian food pattern; hPVG, healthful pro-vegetarian food pattern; uPVG, unhealthful pro-vegetarian food pattern.  ^1.^ Positive indicates that higher consumption of this food group received higher scores. Reverse indicates that higher consumption of this food group received lower scores.  ^2.^ In the hPVG food pattern, whole grains, fruits, vegetables, nuts, legumes, potatoes (boiled), tea, and coffee were considered “healthy plant foods.” Refined grains, fries or chips, fruit juices, sugar-sweetened beverages, and sweets and desserts were considered “unhealthy plant foods.” The gPVG food pattern did not differentiate plant foods as healthy or unhealthy.  ^3.^ In the gPVG food pattern, consumption of whole grains and refined grains were considered as the “grains” group and boiled potatoes and fries or chips were considered as the “potatoes” group. | | | | |
